# Novel Insights into the Role of Chromatin Remodeler MORC2 in Cancer

**DOI:** 10.3390/biom13101527

**Published:** 2023-10-15

**Authors:** Namita Chutani, Sandhya Ragula, Khajamohiddin Syed, Suresh B. Pakala

**Affiliations:** 1 Biology Division, Indian Institute of Science Education and Research (IISER) Tirupati, Mangalam, Tirupati 517 507, India; namitachutani@students.iisertirupati.ac.in; 2 Department of Biochemistry, School of Life Sciences, University of Hyderabad, Hyderabad 500 046, India; 23lbph10@uohyd.ac.in; 3 Department of Biochemistry and Microbiology, Faculty of Science, Agriculture and Engineering, University of Zululand, KwaDlangezwa 3886, South Africa; syedk@unizulu.ac.za

**Keywords:** MORC2, chromatin remodeling, transcription of genes, post-translational modifications, cancer invasion and migration, metabolism

## Abstract

A newly discovered chromatin remodeler, MORC2, is a Microrchidia (MORC) family member. MORC2 acts as a chromatin remodeler by binding to the DNA and changing chromatin conformation using its ATPase domain. MORC2 is highly expressed in a variety of human cancers. It controls diverse signaling pathways essential for cancer development through its target genes and interacting partners. MORC2 promotes cancer cells’ growth, invasion, and migration by regulating the expression of genes involved in these processes. MORC2 is localized primarily in the nucleus and is also found in the cytoplasm. In the cytoplasm, MORC2 interacts with adenosine triphosphate (ATP)-citrate lyase (ACLY) to promote lipogenesis and cholesterogenesis in cancer. In the nucleus, MORC2 interacts with the transcription factor c-Myc to control the transcription of genes involved in glucose metabolism to drive cancer cell migration and invasion. Furthermore, MORC2 recruits on to the promoters of tumor suppressor genes to repress their transcription and expression to promote oncogenesis. In addition to its crucial function in oncogenesis, it plays a vital role in DNA repair. Overall, this review concisely summarizes the current knowledge about MORC2-regulated molecular pathways involved in cancer.

## 1. Introduction

Chromatin remodelers govern various cellular functions, such as transcription, DNA replication, and DNA repair mechanisms, by regulating the access to genomic DNA for the protein complexes that regulate these processes [1,2,3,4]. The chromatin remodelers, typically are ATP-dependent, regulate DNA accessibility to the DNA-binding proteins by ejecting, altering, or repositioning the nucleosomes [2,5,6]. This ensures cellular identity determination and normal cell function [2]. The deregulated expression of chromatin remodelers leads to altered chromatin structure and gene expression, which promotes cancer and other diseases [1,7,8,9,10,11,12]. Therefore, knowledge about the effects and significance of the chromatin remodeling families and complexes is emerging.

One of the emerging chromatin remodeling family members in eukaryotes is Microrchidia (MORC). MORCs are widely expressed and play an important role in chromatin remodeling and epigenetic control [13]. For the first time, Watson and others discovered an insertional mutation (morcTgN (Tyr)1Az) in Morc1 in mice, and they demonstrated that this Morc1 mutation is responsible for inhibiting spermatogenesis and germ cell death [14]. While Morc -/- female mice were normal, Morc -/- male mice were found to be infertile with diminished testicular mass [14]. This suggests that the MORC gene has specific functions and is essential for male gametogenesis. Five MORC family members were identified in humans: MORC1, MORC2, MORC3, MORC4, and a divergent SMCHD1 (Structural Maintenance of Chromosomes Flexible Hinge Domain 21 containing 1) [13]. MORC family members have different biological activities but a typical domain architecture (CW-type zinc finger, GHKL-ATPase, and coiled-coil domains) [15]. These family members were further divided into two subfamilies based on the structure of the ZF-CW domain, where MORC1 and MORC2 were placed in subfamily-I and MORC3 and MORC4 were placed in subfamily-IX [16].

The human MORC family members express differentially in various tissues, supporting multiple biological functions. For instance, MORC1 expression has been detected in thymocytes, embryonic stem cells, and reproductive tissues such as the testis and is majorly involved in spermatogenesis [17,18]. MORC2 protein is expressed ubiquitously [17,18]. Low MORC2 protein expression levels have been observed in the hematological, immunological, secretory, and reproductive systems, whereas the liver and kidney have shown strong MORC2 protein expression [18]. MORC2 was shown to have potential role in gene transcription, DNA damage repair process, and adipogenic differentiation (18). MORC3 protein localization is restricted to the blood, immunological, secretory, and reproductive systems. MORC3 is involved in the transcriptional repression of genes and regulating calcium and bone homeostasis [18]. Meanwhile, MORC4 expression has been found in the placenta and testis and is involved in spermatogenesis [17,18].

**Figure 1:**
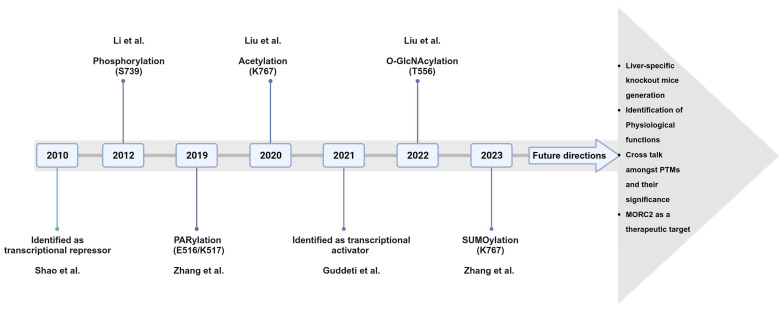
Timeline of major advances in MORC2-mediated transcriptional processes and MORC2 post-translational modifications. Shao et al. discovered that MORC2 has transcriptional repressor activity, which reduces the expression of CAIX, and is the first transcriptional target gene of MORC2 [19]. Li et al. identified that, in response to DNA damage, p21-activated kinase 1 (PAK1) phosphorylates MORC2 at serine 739, which helps to promote chromatin remodeling in order to recruit DNA repair proteins to the site of DNA damage [20]. Zhang et al. found that the chromatin-associated enzyme poly (ADP-ribose) polymerase 1 (PARP1) interacts with and PARylates MORC2 at E516/K517 to activate its ATPase and chromatin remodeling function in response to DNA damage [21]. Liu et al. identified that the acetyltransferase NAT10 acetylates MORC2 at lysine 767 (K767Ac) in response to DNA-damaging chemotherapeutic drugs and ionizing radiation [22]. Guddeti et al. identified that MORC2 interacts with a transcriptional factor c-Myc and gets recruited onto LDHA promoter in order to drive its transcription [23]. Liu et al. discovered that transforming growth factor-β1 (TGF-β1) stimulates MORC2 O-GlcNacylation at threonine 556 (T556) by an enzyme O-GlcNAc transferase (OGT) to promote breast cancer cell migration and invasion [24]. Zhang et al. reported that small ubiquitin-like modifier 1 (SUMO1) and SUMO 2/3,SUMOylates MORC2 at lysine 767 to promote DNA repair [25].

MORC family members are highly dysregulated in various clinical conditions, including cancers, neurological illnesses, and metabolic bone diseases [18]. Among the MORC family members, MORC2 has a unique position due to its overexpression or deregulated expression in a wide range of human cancers and its function in the transcriptional regulation of genes involved in oncogenesis, DNA damage response, promoting cancer cell invasion, migration, cancer metabolism, metastasis, and chemoresistance (Figure 1). In this review, we emphasize the current developments in our knowledge of MORC2’s role as a transcriptional regulator, its post-translational modifications (PTMs), and its functions in regulating hallmarks of cancer.

## 2. MORC2 Domain Organization and Structure

**Figure 2:**
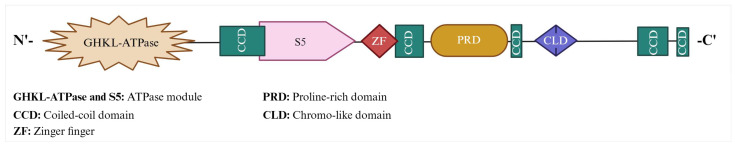
MORC2 protein structure and domain organization. MORC2 contains GHKL domain (1–278 a.a), S5 domain (323–469 a.a.), ZF-CW (ZF) domain (490–544 a.a.), PRD (601–734 a.a.), CLD (790–854 a.a.), and five CCDs (CCD1 (282–362 a.a.): CCD2 (547–584 a.a.); CCD3 (741–761 a.a.); CCD4 (966–1016 a.a.), and CCD5 (1024–1032 a.a.)). ATPase domain (1–469 a.a.) includes both GHKL and S5.

The human MORC2 gene is at position q12.2 on the reverse strand of chromosome 22. So far, two functional protein coding MORC2 isoforms have been identified. MORC2 isoform-1 and 2 contain 1032 and 970 amino acids (a.a.), respectively. The transcript variant that codes for 970 a.a. isoform 2 (Uniprot ID: Q9Y6X9-2) has an additional exon, which results in the use of an in-frame downstream start codon, compared to the transcript variant 1 that codes for 1032 a.a. isoform (Uniprot ID: Q9Y6X9-1). This difference results in a shorter N-terminus compared to isoform-1. Although both MORC2 isoforms have different numbers of amino acids, their ATPase domain remains functionally active. The basic structure of MORC2 consists of a GHKL-ATPase (gyrase; heat shock protein, HSP 90; histidine kinase; DNA mismatch repair, Mut L) domain, zinc finger (ZF)-CW domain, coiled-coil domains, proline-rich domain (PRD), and chromo-like domain (CLD) (Figure 2).

The GHKL-ATPase domain is necessary for MORC2 to function as an ATPase, and as a result of this function, MORC2 regulates chromatin remodeling in response to DNA damage [20]. The transcription activation suppressor (TASOR), M-phase phosphoprotein 8 (MPP8), and periphilin are components of the Human Silencing Hub (HUSH) complex [26]. Periphilin binds to RNA and directs HUSH to its target loci, where HUSH recruits the Su(var)3-9, enhancer of zeste and trithorax (SET) domain bifurcated histone lysine methyltransferase 1 (SETDB1), MORC2, and nuclear exosome-targeting (NEXT) complex. SETDB1 deposits histone 3 lysine 9 trimethylation (H3K9me3). MPP8 recognizes H3K9me3 and allows the HUSH complex to bind at the locus to repress the gene transcription. It has been demonstrated that MORC2′s ATPase domain is necessary for the functions of the HUSH complex [26]. The ZF-CW domain is a reader domain with a comparatively short motif of 50–60 amino acids. This domain contains four conserved cysteines (C) and two conserved tryptophan (W) residues, which can bind to zinc to form finger-like projections, enabling them to interact with the target genes that participate in processes such as the recognition of methylated histones, chromatin remodeling [27], epigenetic control, and embryonic development [28]. However, functionally, the MORC2 ZF-CW domain is not well characterized. The coiled-coil domain plays a significant role in nucleic acid binding, protein stability, assembly, and protein–protein interactions [29,30,31,32,33]. The MORC2 protein includes five coiled-coil domains in total, allowing MORC2 to dimerize. The dimerization of MORC2 is a critical event in the initiation of DNA repair signaling in response to DNA damage [30]. Although MORC2 has several coiled-coil domains, each domain interacts with different partners for a specific function. The coiled-coil domain 1 of MORC2 interacts with chromatin by binding to DNA, whereas the C-terminal coiled-coil domains aid in dimerization, which is important for its contributing functions, such as DNA-damage repair and cell survival [30,34]. In addition to the common MORC protein domains, MORC2 has a PRD (proline-rich domain) and a CLD (chromo-like domain) [13]. The PRD domain of MORC2 interacts with a cadherin-associated protein, catenin delta 1 (CTNND1), to promote tumor invasion and metastasis [35]. The MORC2-CLD function is still unknown. Additionally, the MORC2 protein also has one putative bipartite and two putative monopartite Nuclear Localization Signals (NLSs), which lie between 657 and 781 a.a., and a further Nuclear Export Signal (NES), which lies between 481 and 657 a.a. Both the NLSs and NES facilitate MORC2 to become localized in the nucleus and cytoplasm, respectively. Wang et al. experimentally showed that most of the MORC2 protein is located in the nucleus rather than in the cytoplasm and concluded that this might be due to the fact that NLSs are more prevalent than NESs [36]. External cues, such as stress, growth factors, cytokines, etc., regulate the localization of MORC2 [17,18,20,35]. However, the shuttling of MORC2 between intracellular spaces is still an unventured question to solve.

## 3. MORC2 Is a Transcriptional Regulator

**Figure 3:**
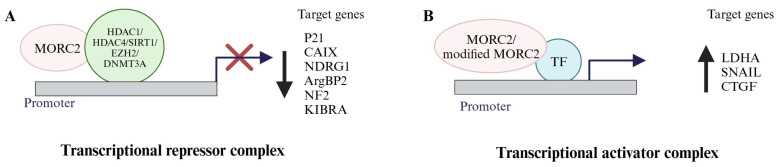
MORC2 is a transcriptional regulator. (**A**) MORC2 acts as a transcriptional repressor of target genes (p21, CAIX, NDRG1, ArgBP2, NF2, and KIBRA) by interacting with HDACs/SIRT1/EZH2/DNMT3A. (**B**) Either MORC2 or modified MORC2 acts as a transcriptional activator of target genes (LDHA, SNAIL, and CTGF) via interacting with transcriptional factors or might be interacting with the chromatin.

In response to upstream signaling molecules such as growth factors, cytokines, metabolites and radiation, chromatin remodelers, and chromatin modifiers regulate the expression of target genes implicated in cancer progression and metastasis. Due to its ATPase activity, MORC2 changes the conformation of the chromatin in an energy-dependent manner [34]. Additionally, MORC2 associates with transcription factors or histone deacetylases (HDACs) to coordinate the expression of target genes implicated in metabolism and cancer progression (Figure 3). As shown below, it acts as an activator or a repressor to control the transcription of target genes involved in cancer.

### 3.1. MORC2 Acts as a Transcription Repressor

MORC2 contributes to target gene transcription because of the CW zinc finger motif. Shao et al. identified carbonic anhydrase IX (CAIX) as the target gene of MORC2 using DNA microarray hybridization and northern and western blot analyses [19]. CAIX was the first target gene of MORC2 to be discovered [19]. In various human cancers, including those of the cervix, breast, head and neck, lung, and brain, overexpression of CAIX has been noted [37]. CAIX promotes tumorigenesis and invasion upon CAIX overexpression [38]. Furthermore, the overexpression of CAIX is also associated with poor prognoses [39]. According to Shao et al.’s research [19], MORC2 and HDAC4 are recruited to the CAIX promoter, repressing CAIX gene transcription. Based on these observations, it was concluded that MORC2 binds to the CAIX promoter first, then recruits HDAC4, which decreases the histone H3 acetylation status of the CAIX promoter, causing the chromatin to close and suppressing CAIX expression [19]. From 2010 onwards, several studies provided insights on MORC2 transcriptional repressor functions, where MORC2 decreased the expression of p21Waf/Cip1 (p21), arg-binding protein 2 (ArgBP2), N-myc Downstream-Regulated Gene 1 (NDRG1), neurofibromatosis 2 (NF2), and kidney- and brain-expressed protein (KIBRA) (Figure 3). The CDK inhibitor p21 is a crucial regulator of cell survival and proliferation [40]. Zhang et al. [41] and Ou, Kepeng et al. [42] found that MORC2 expression is inversely correlated with p21 expression in individuals with gastric and colorectal cancers, respectively. Zhang et al. also discovered that MORC2 and HDAC1 are recruited onto the p21 promoter to repress its expression in a p53-independent manner. The MORC2-mediated p21 repression enhances cell cycle progression, resulting in alterations in the cell cycle, as seen by a decreased percentage of G1 phase cells and an increased percentage of S and G2/M phase cells. Further, they also discovered that MORC2 enhances gastric cancer cell proliferation by controlling cell cycle progression via p21 suppression [41]. Kepeng et al. analyzed the RNA sequence and clinical data from colorectal cancer (CRC) patients in the TCGA data base, and they identified elevated MORC2 levels. By performing CCK8 and colony formation assays under the conditions of MORC2 knockdown, they found that MORC2 has a role in CRC proliferation. They observed a positive correlation between the expression levels of p53 and p21 along with the proinflammatory cytokines IL-6 and IL-8 responsible for cellular senescence via decreasing the expression of HDAC4. Based on these findings, they concluded that increased MORC2 plays a key role in CRC tumorigenesis by controlling cellular senescence [42]. ArgBP2 controls the reorganization of the actin cytoskeleton [43]. Tong et al. [44] discovered low expression levels of ArgBP2 mRNA in gastric cancer tumor samples. They identified that ArgBP2 overexpression inhibits the proliferation, invasion, and migration of gastric cancer cells. They identified ArgBP2 as a target gene of MORC2. They demonstrated that MORC2 enhances the enhancer of zeste homolog 2 (EZH2), a polycomb repressive complex 2 (PRC2) subunit recruitment onto the ArgBP2 promoter, which promotes tri-methylation of H3K27 and thereby represses ArgBP2 transcription. Due to this, there is low-level expression of ArgBP2 in gastric cancer cells, which is responsible for gastric cancer cell proliferation [44]. Alternatively, MORC2 also represses ArgBP2 transcription by enhancing the recruitment of heat shock factor protein 1 (HSF1) and PRC2 onto the ArgBP2 promoter [45]. NDRG1 is a metastasis suppressor and prognostic colorectal cancer (CRC) biomarker. Liu et al. [46] identified that MORC2 downregulates NDRG1 expression in CRC cells and observed a negative correlation between MORC2 and NDRG1 in CRC patients. They found that MORC2 interacts with sirtuin1 (SIRT1) and gets recruited onto the NDRG1 promoter to repress the transcription and expression of NDRG1. Furthermore, the increased expression levels of MORC2 and decreased expression of NDRG1 are associated with lymph node metastasis in CRC samples [46]. In addition, Wang et al. [47] reported that MORC2, along with DNA methyltransferase 3A (DNMT3A), represses NF2 and KIBRA expression by recruiting onto their promoters to facilitate tumorigenesis and cancer stemness in hepatocellular carcinoma.

### 3.2. MORC2 Acts as a Transcription Activator

Based on the signal, MORC2 protein, in association with transcription factors or coactivator complexes, regulate/or activate the target gene’s transcription in a promoter context-dependent manner. So far, MORC2 has been found to act as a transcriptional coactivator for activating the transcription of the genes lactate dehydrogenase A (LDHA), Connective Tissue Growth Factor (CTGF), and Snail Family Transcriptional Repressor 1 (SNAIL) [23,24]. LDHA is the key enzyme involved in this process, where it converts pyruvate to lactate even in the presence of oxygen in cancer cells. Guddeti et al. [23] demonstrated that MORC2 is a glucose-inducible gene and a transcriptional target gene of c-Myc. Cancer cells treated with a high glucose concentration induce the expression of MORC2, which interacts with c-Myc and gets recruited onto the LDHA promoter to increase the transcription of LDHA. Additionally, they demonstrated that the MORC2–c-Myc–LDHA signaling axis contributes to the migration of breast cancer cells [23]. This was the first study to describe MORC2’s role as a transcription activator in breast cancer cells in association with the transcription factor c-Myc.

Further, Liu et al. [24] discovered that the Transforming Growth Factor β1 (TGF-β1) target genes CTGF and SNAIL, are also transcriptionally activated by MORC2 (O-GlcNAcylated MORC2). TGF-β1 signaling aids in the progression of breast cancer by controlling the transcription of the genes SNAIL and CTGF, which have a proven role in tumor metastasis [48,49]. SNAIL is a potent inducer of breast cancer invasion and metastasis and a master regulator of TGF-β1-driven epithelial–mesenchymal transitions [50,51]. The migratory, invasive, metastatic, and angiogenic processes in human breast cancer cells are all significantly influenced by CTGF [52,53]. Liu et al. identified that O-GlcNActransferase (OGT) interacts with MORC2 and promotes the O-GlcNAcylation of MORC2. They found that glucose induces MORC2 O-GlcNAcylation in a dose-dependent manner in MCF-7 and T47D cells treated with glucose. Further, they tried to understand the role of TGF-β1 on MORC2 O-GlcNAcylation, as the growth factor influences the O-GlyNAcylation of proteins. They found that TGF-β1 induces the O-GlcNAcylation of MORC2 via increasing the stability of glutamine-fructose-6-phosphate aminotransferase (GFAT), which is needed for the synthesis of OGT. In addition, they noticed that O-GlcNAcylated MORC2 regulates the expression of SNAIL and CTGF by recruiting onto the promoters of SNAIL (−151 to −476) and CTGF (-1035 to -1235 and -1534 to -1925). Additionally, the knockdown of GFAT, SNAIL, or CTGF compromised the ability of TGF-β1-induced MORC2 O-GlcNAcylation-mediated breast cancer cell migration and invasion. They recognized that breast cancer patients with high-level expressions of MORC2, OGT, SNAIL, and CTGF had poor prognosis [24]. Overall, these findings establish that MORC2 regulates the TGF-β1 target genes SNAIL and CTGF transcriptionally [24].

## 4. MORC2 Post-Translational Modifications and Their Significance

**Figure 4:**
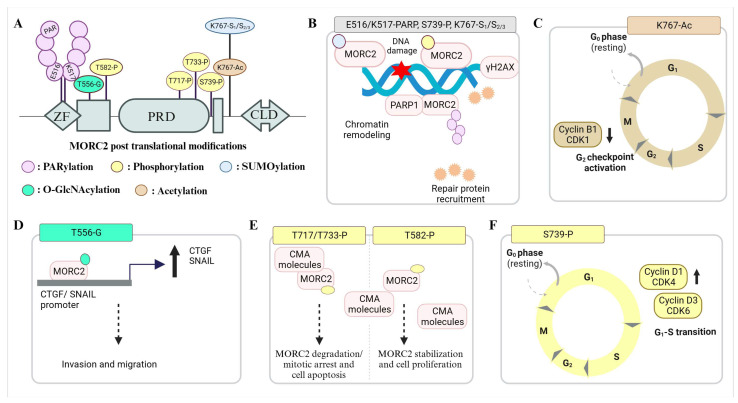
MORC2 post-translational modifications and their functional significance. (**A**) MORC2 undergoes post-translational modifications such as PARylation (E516, K517), phosphorylation (T582, T717, T733, S739), SUMOylation (K767), O-GlcNacylation (T556), and acetylation (K767). (**B**) In response to DNA damage, MORC2 is either phosphorylated or SUMOylated and recruits onto chromatin to open, allowing the PARP and DNA repair proteins to bind and repair the double-stranded breaks. (**C**) Ionizing radiation and chemotherapy drugs that damage DNA promote MORC2 acetylation. The acetylated MORC2 transcriptionally inhibits the expression of CDK1 and Cyclin B1 to promote DNA-damage-induced G2 checkpoint activation. (**D**) O-GlcNAcylated MORC2 increases the expression of SNAIL and CTGF and thereby promotes breast cancer cell invasion and migration. (**E**) The model depicts MORC2-phosphorylation-dependent stabilization and destabilization events. Phosphorylation of MORC2 (T717, T733) by paclitaxel (PTX) and vincristine (VCR) activated CDK1 leads to its destruction via CMA pathway. Phosphorylation of MORC2 at T582 by GPER1-activated PRKACA reduces its ability to interact with CMA machinery components (HSPA8 and LAMP2A) and thereby increases its stability and oncogenic potential. (**F**) The expression of the Cyclin D1-CDK4 and Cyclin D3-CDK6 complexes is increased by PAK1-mediated phosphorylation of MORC2 at S677 (S739 in Isoform 1), which facilitates the transition of the gastric cell cycle from G1 to S.

Post-translation modification (PTM) plays an important role in regulating distribution of MORC2 function(s) in DNA damage response and cancer development. The first PTM discovered on a MORC2 protein was phosphorylation (Figure 4). Since then, diverse PTMs on MORC2 have been studied spanning the post-ATPase domain region. Upon genotoxic stress stimulations, MORC2 acts as a substrate for p21 activated kinase 1 (PAK1), N-acetyltransferase (NAT10), poly (ADP Ribose) polymerase 1 (PARP1), and tripartite motif-containing 28 (TRIM28) and becomes phosphorylated, acetylated, poly (ADP-ribosyl)ated, and SUMOylated [20,21,22,25]. DNA damage triggers MORC2 phosphorylation by PAK1, and further phosphorylated MORC2 recruits onto DNA damage sites and induces histone H2AX phosphorylation to facilitate chromatin-remodeling-mediated DNA repair [20]. MORC2, NAT10, and PARP1 also play a crucial role in genome surveillance as a complex [21,22,54]. NAT10 acetylates MORC2 at the lysine 767 residue (K767Ac). Acetylated MORC2 promotes cell survival by reducing the phosphorylation of the 11th threonine residue of the histone H3 protein (H3T11P), transcriptional repression of CDK1 and Cyclin B1, and subsequent G2 DNA damage checkpoint activation [22]. MORC2 acetylation and its role in DNA damage is further regulated by the PARP1-facilitated nucleoplasm translocation of the NAT10 enzyme [54]. At DNA damage sites, MORC2 is recruited by the PARP1 enzyme and gets PARylated [21]. MORC2 PARylation further facilitates the increased accessibility of damaged chromatin and, in turn, promotes PAR-dependent DNA repair by stabilizing the PARP1 protein from ubiquitination [21]. The response of DNA damage and MORC2 post-translational modifications is intriguingly regulated in a time- and damage-dose-dependent manner. At the late stages of DNA damage, MORC2 is SUMOylated by TRIM28 at K767, and the SUMOylated MORC2 activates DNA repair kinases, thereby promoting chromatin accessibility, DNA repair, and chemoresistance [25]. The MORC2 phosphorylation at Ser739 and acetylation at Lys767 regulate the SUMOylaiton status at Lys767 and exhibit negative crosstalk upon damage, respectively [25].

In addition to the role of MORC2 in DNA repair and response, MORC2 also plays a quintessential role in regulating cancer cell proliferation, such as (i) PAK1-mediated MORC2 phosphorylation, which is required to promote gastric cancer cell proliferation by facilitating G1–S transitions and (ii) O-GlcNAcylated MORC2, which is induced by TGF-β1 treatment, recruits TGF-β1 target genes (CTGF and SNAIL) and plays a requisite role in their transcriptional activation by an unknown mechanism, promoting breast cancer invasion and migration [24]. E2- and ESR1-targeting drugs mediate breast cancer proliferation by preventing MORC2 lysosomal degradation via chaperon-mediated autophagy [55]. An estrogenic receptor (GPER1) activates protein kinase cAMP-activated catalytic subunit alpha (PRKACA), which in turn phosphorylates MORC2 at Thr582 [55]. Phosphorylation reduces interactions with CMA machinery molecules (HSPA8 and LAMP2A), enhancing its stability and oncogenic activity [55]. Hu et al. identified that the microtubule-targeting drugs paclitaxel (PTX) and vincristine (VCR) promote CDK1-mediated MORC2 phosphorylation (T717 and T733), which causes MORC2 degradation through a chaperone-mediated pathway. Upon treatment with PTX and VCR, MORC2 degradation helps in prolonged mitotic arrest and the death of cancer cells [56]. The dynamic states of post-translationally modified MORC2 play a key role in mediating its protein turnover, resistance to DNA-damaging agents, cell survival, checkpoint activation, and genome integrity by chromatin remodeling and recruiting repair machinery.

## 5. MORC2 Role in Cancer

Nearly all the human cells and tissues were found to express the MORC2 protein [17,18]. MORC2 expression is very high in cancer cells compared to normal cells [18]. Several cancers, including breast, lung, liver, prostate, stomach, colon, ovarian, pancreatic, and endometrial, have dysregulated MORC2 expression [57,58]. This increased expression of MORC2 is linked to cancer cell proliferation, metabolism, invasion, migration, and metastasis and confers chemotherapy resistance [15,23,24,25,35,42,46,55,57,58,59,60,61,62,63,64]. Furthermore, increased MORC2 expression levels were linked to poor prognosis and unfavorable clinical outcomes in non-small cell lung and breast cancer [58]. Notably, MORC2 interacts with many proteins to control the expression of its downstream targets, which promote the hallmarks of cancer or directly modulate the hallmarks of cancer by regulating the transcription of its target genes [15,57].

MORC2 is overexpressed in colorectal cancer, and by controlling cellular senescence, it promotes tumorigenesis [42]. Saroha et al.’s [62] study revealed that MORC2 regulates β-catenin expression and function by modulating the phosphorylation of AKT. They noticed reduced proliferation and migration of MORC2-overexpressing breast cancer cells upon β-catenin inhibition. They concluded from these findings that MORC2 regulates β-catenin signaling to support breast cancer cell proliferation and migration [62]. By controlling the PTEN/PI3K/AKT signaling pathway, MORC2 promotes glioma cell proliferation, migration, invasion, and epithelial–mesenchymal transition (EMT) [60]. Additionally, Wang et al. demonstrated that PAK1-mediated phosphorylation of MORC2 promotes the growth and development of gastric cancer [65]. Wang et al. also discovered that MORC2, in collaboration with DNA methyltransferase 3A (DNMT3A), regulates the expression of NF2 and KIBRA to support carcinogenesis and cancer stemness in hepatocellular carcinoma [47]. Further, MORC2 interacts with EZH2 or HSF1 to control the expression of ArgBP2, which promotes the proliferation, invasion, and migration of cancer cells [44,45]. Furthermore, MORC2–SIRT1 complex suppresses NDRG1 expression to encourage metastasis in colorectal cancer [46]. Liao et al. showed that MORC2 interacts with catenin delta 1 (CTNND1) to promote invasion and metastasis in breast cancer [35]. Zhang et al. found that the mutant MORC2 (M276I) stimulates the splicing-based switch of CD44 from the epithelial isoform to the mesenchymal isoform in association with hnRNPM (heterogeneous nuclear ribonucleoprotein M), and this resulted in epithelial to mesenchymal transition [66].

The Warburg effect is a process whereby rapidly growing cancer cells consume large amounts of glucose and metabolize it by aerobic glycolysis [67,68,69]. It was discovered that MORC2 alters the metabolism of cancer cells by regulating glucose metabolism and lipogenesis [23,70,71]. The expression of MORC2 is induced with high glucose concentration in cancer cells. MORC2 then interacts with the transcription factor c-Myc and is recruited to the LDHA promoter to control the expression of LDHA, which is responsible for the migration of breast cancer cells [23]. Additionally, Guddeti et al. demonstrated that the MORC2–c-Myc–MAX complex regulates the expression of glycolytic enzymes and, thereby, breast cancer cell proliferation and migration [70]. Furthermore, Sanchez-Solana et al. reported that MORC2 interacts with adenosine triphosphate (ATP)-citrate lyase (ACLY), an enzyme that catalyzes the production of acetyl-CoA, which is a key metabolite for lipogenesis, cholesterogenesis, and histone acetylation [71]. Su et al. also showed that circDNM3OS increased MORC2 expression via sponging miR-145-5p, promoting glutamine metabolism and cholangiocarcinoma (CCA) [72].

Recently, several studies showed that MORC2 contributes to chemoresistance. By examining the expression levels of apoptosis-related proteins under MORC2 knockdown conditions, Pan et al. identified the mechanism through which MORC2 influences chemoresistance. The release of mitochondrial cytochrome c into the cytosol and the cleavage of caspase-9, caspase-3, and PARP were caused by MORC2 knockdown. These findings led them to the conclusion that MORC2 might be involved in chemoresistance [64]. In response to DNA damage, the SUMOylation of MORC2 orchestrates chromatin remodeling and DNA repair and promotes chemoresistance in breast cancer [25]. Yang et al. discovered that the phosphorylation of MORC2 in response to estrogen and antiestrogens inhibits CMA-mediated lysosomal degradation and plays a significant role in estrogen-induced proliferation and resistance to antiestrogen therapies in breast cancer [55]. The acetylation of MORC2 by NAT10 regulates cell-cycle check point control and resistance to DNA-damaging chemotherapy and radiotherapy in breast cancer [22]. Liao et al.’s investigation revealed that high MORC2 expression is a sign of worse neoadjuvant chemotherapy efficacy in triple-negative breast cancer (TNBC) [59].

## 6. Conclusions

A key member of the MORC chromatin remodeler family of proteins, MORC2 controls the expression of target genes linked to human cancer and neurological diseases. MORC2 biology has been the subject of significant scholarly investigation for approximately 20 years since its discovery. It is well known that MORC2 is involved in the control of several cancer-related functions. In this review, we outlined several transcriptional regulatory functions of MORC2 and post-translational modifications of MORC2 and their relevance to cancer. Overall, it is widely known that MORC2 plays a role in the DNA damage response and DNA repair mechanisms. It is exciting to know that MORC2 plays a crucial role in cancer metabolism, specifically in lipogenesis and glucose metabolism. Additionally, MORC2-driven signaling pathways support cell proliferation, invasion, migration, and metastasis. However, MORC2’s precise physiological activities in normal tissues are still unclear. The generation of MORC2 knockout mice, especially MORC2 liver knockout mice, is urgently required to comprehend the functional significance of MORC2 in metabolism and metastasis, as we and other studies reported that MORC2 has a role in glucose, glutamine, and lipid metabolism in cancer. Further, we are also interested in understanding MORC2 regulatory networks to develop MORC2-targeted anticancer therapeutics.

## References

[1] Nair S.S., Kumar R. (2012). Chromatin remodeling in cancer: A gateway to regulate gene transcription. Mol. Oncol..

[2] Reyes A.A., Marcum R.D., He Y. (2021). Structure and Function of Chromatin Remodelers. J. Mol. Biol..

[3] Tyagi M., Imam N., Verma K., Patel A.K. (2016). Chromatin remodelers: We are the drivers!!. Nucleus.

[4] Zhang P., Torres K., Liu X., Liu C.G., Pollock R.E. (2016). An Overview of Chromatin-Regulating Proteins in Cells. Curr. Protein Pept. Sci..

[5] Clapier C.R., Iwasa J., Cairns B.R., Peterson C.L. (2017). Mechanisms of action and regulation of ATP-dependent chromatin-remodelling complexes. Nat. Rev. Mol. Cell Biol..

[6] Magana-Acosta M., Valadez-Graham V. (2020). Chromatin Remodelers in the 3D Nuclear Compartment. Front. Genet..

[7] Cairns B.R. (2001). Emerging roles for chromatin remodeling in cancer biology. Trends Cell Biol..

[8] Cairns B.R. (2007). Chromatin remodeling: Insights and intrigue from single-molecule studies. Nat. Struct. Mol. Biol..

[9] Di Croce L. (2005). Chromatin modifying activity of leukaemia associated fusion proteins. Hum. Mol. Genet..

[10] Ellis L., Atadja P.W., Johnstone R.W. (2009). Epigenetics in cancer: Targeting chromatin modifications. Mol. Cancer Ther..

[11] Mitelman F., Johansson B., Mertens F. (2007). The impact of translocations and gene fusions on cancer causation. Nat. Rev. Cancer.

[12] Vaicekauskaite I., Sabaliauskaite R., Lazutka J.R., Jarmalaite S. (2022). The Emerging Role of Chromatin Remodeling Complexes in Ovarian Cancer. Int. J. Mol. Sci..

[13] Chutani N., Singh A.K., Kadumuri R.V., Pakala S.B., Chavali S. (2022). Structural and Functional Attributes of Microrchidia Family of Chromatin Remodelers. J. Mol. Biol..

[14] Watson M.L., Zinn A.R., Inoue N., Hess K.D., Cobb J., Handel M.A., Halaban R., Duchene C.C., Albright G.M., Moreadith R.W. (1998). Identification of morc (*microrchidia*), a mutation that results in arrest of spermatogenesis at an early meiotic stage in the mouse. Proc. Natl. Acad. Sci. USA.

[15] Guddeti R.K., Chutani N., Pakala S.B. (2021). MORC2 Interactome: Its Involvement in Metabolism and Cancer. Biophys. Rev..

[16] Perry J., Zhao Y. (2003). The CW domain, a structural module shared amongst vertebrates, vertebrate-infecting parasites and higher plants. Trends Biochem. Sci..

[17] Hong G., Qiu H., Wang C., Jadhav G., Wang H., Tickner J., He W., Xu J. (2017). The Emerging Role of MORC Family Proteins in Cancer Development and Bone Homeostasis. J. Cell Physiol..

[18] Wang H., Zhang L., Luo Q., Liu J., Wang G. (2021). MORC protein family-related signature within human disease and cancer. Cell Death Dis..

[19] Shao Y., Li Y., Zhang J., Liu D., Liu F., Zhao Y., Shen T., Li F. (2010). Involvement of histone deacetylation in MORC2-mediated down-regulation of carbonic anhydrase IX. Nucleic Acids Res..

[20] Li D.Q., Nair S.S., Ohshiro K., Kumar A., Nair V.S., Pakala S.B., Reddy S.D., Gajula R.P., Eswaran J., Aravind L. (2012). MORC2 signaling integrates phosphorylation-dependent, ATPase-coupled chromatin remodeling during the DNA damage response. Cell Rep..

[21] Zhang L., Li D.Q. (2019). MORC2 regulates DNA damage response through a PARP1-dependent pathway. Nucleic Acids Res..

[22] Liu H.Y., Liu Y.Y., Yang F., Zhang L., Zhang F.L., Hu X., Shao Z.M., Li D.Q. (2020). Acetylation of MORC2 by NAT10 regulates cell-cycle checkpoint control and resistance to DNA-damaging chemotherapy and radiotherapy in breast cancer. Nucleic Acids Res..

[23] Guddeti R.K., Thomas L., Kannan A., Karyala P., Pakala S.B. (2021). The chromatin modifier MORC2 affects glucose metabolism by regulating the expression of lactate dehydrogenase A through a feed forward loop with c-Myc. FEBS Lett..

[24] Liu Y.Y., Liu H.Y., Yu T.J., Lu Q., Zhang F.L., Liu G.Y., Shao Z.M., Li D.Q. (2022). O-GlcNAcylation of MORC2 at threonine 556 by OGT couples TGF-beta signaling to breast cancer progression. Cell Death Differ..

[25] Zhang F.L., Yang S.Y., Liao L., Zhang T.M., Zhang Y.L., Hu S.Y., Deng L., Huang M.Y., Andriani L., Ma X.Y. (2023). Dynamic SUMOylation of MORC2 orchestrates chromatin remodelling and DNA repair in response to DNA damage and drives chemoresistance in breast cancer. Theranostics.

[26] Tchasovnikarova I.A., Timms R.T., Douse C.H., Roberts R.C., Dougan G., Kingston R.E., Modis Y., Lehner P.J. (2017). Hyperactivation of HUSH complex function by Charcot-Marie-Tooth disease mutation in MORC2. Nat. Genet..

[27] Ciquier G., Azzi M., Hebert C., Watkins-Martin K., Drapeau M. (2021). Assessing the quality of seven clinical practice guidelines from four professional regulatory bodies in Quebec: What’s the verdict?. J. Eval. Clin. Pract..

[28] Liu Y., Tempel W., Zhang Q., Liang X., Loppnau P., Qin S., Min J. (2016). Family-wide Characterization of Histone Binding Abilities of Human CW Domain-containing Proteins. J. Biol. Chem..

[29] Yoshinaka T., Kosako H., Yoshizumi T., Furukawa R., Hirano Y., Kuge O., Tamada T., Koshiba T. (2019). Structural Basis of Mitochondrial Scaffolds by Prohibitin Complexes: Insight into a Role of the Coiled-Coil Region. iScience.

[30] Xie H.Y., Zhang T.M., Hu S.Y., Shao Z.M., Li D.Q. (2019). Dimerization of MORC2 through its C-terminal coiled-coil domain enhances chromatin dynamics and promotes DNA repair. Cell Commun. Signal..

[31] Cristie-David A.S., Chen J., Nowak D.B., Bondy A.L., Sun K., Park S.I., Banaszak Holl M.M., Su M., Marsh E.N.G. (2019). Coiled-Coil-Mediated Assembly of an Icosahedral Protein Cage with Extremely High Thermal and Chemical Stability. J. Am. Chem. Soc..

[32] Matityahu A., Onn I. (2018). A new twist in the coil: Functions of the coiled-coil domain of structural maintenance of chromosome (SMC) proteins. Curr. Genet..

[33] Terawaki S., Yoshikane A., Higuchi Y., Wakamatsu K. (2015). Structural basis for cargo binding and autoinhibition of Bicaudal-D1 by a parallel coiled-coil with homotypic registry. Biochem. Biophys. Res. Commun..

[34] Douse C.H., Bloor S., Liu Y., Shamin M., Tchasovnikarova I.A., Timms R.T., Lehner P.J., Modis Y. (2018). Neuropathic MORC2 mutations perturb GHKL ATPase dimerization dynamics and epigenetic silencing by multiple structural mechanisms. Nat. Commun..

[35] Liao X.H., Zhang Y., Dong W.J., Shao Z.M., Li D.Q. (2017). Chromatin remodeling protein MORC2 promotes breast cancer invasion and metastasis through a PRD domain-mediated interaction with CTNND1. Oncotarget.

[36] Wang G.L., Wang C.Y., Cai X.Z., Chen W., Wang X.H., Li F. (2010). Identification and expression analysis of a novel CW-type zinc finger protein MORC2 in cancer cells. Anat. Rec..

[37] Tafreshi N.K., Lloyd M.C., Bui M.M., Gillies R.J., Morse D.L. (2014). Carbonic anhydrase IX as an imaging and therapeutic target for tumors and metastases. Subcell. Biochem..

[38] Chen J., Rocken C., Hoffmann J., Kruger S., Lendeckel U., Rocco A., Pastorekova S., Malfertheiner P., Ebert M.P. (2005). Expression of carbonic anhydrase 9 at the invasion front of gastric cancers. Gut.

[39] Driessen A., Landuyt W., Pastorekova S., Moons J., Goethals L., Haustermans K., Nafteux P., Penninckx F., Geboes K., Lerut T. (2006). Expression of carbonic anhydrase IX (CA IX), a hypoxia-related protein, rather than vascular-endothelial growth factor (VEGF), a pro-angiogenic factor, correlates with an extremely poor prognosis in esophageal and gastric adenocarcinomas. Ann. Surg..

[40] Besson A., Dowdy S.F., Roberts J.M. (2008). CDK inhibitors: Cell cycle regulators and beyond. Dev. Cell.

[41] Zhang Q., Song Y., Chen W., Wang X., Miao Z., Cao L., Li F., Wang G. (2015). By recruiting HDAC1, MORC2 suppresses p21 Waf1/Cip1 in gastric cancer. Oncotarget.

[42] Ou K., Li Y., Long Y., Luo Y., Tang D., Chen Z. (2022). Inhibition of MORC2 Mediates HDAC4 to Promote Cellular Senescence through p53/p21 Signaling Axis. Molecules.

[43] Roignot J., Soubeyran P. (2009). ArgBP2 and the SoHo family of adapter proteins in oncogenic diseases. Cell Adhes. Migr..

[44] Tong Y., Li Y., Gu H., Wang C., Liu F., Shao Y., Li J., Cao L., Li F. (2015). Microchidia protein 2, MORC2, downregulates the cytoskeleton adapter protein, ArgBP2, via histone methylation in gastric cancer cells. Biochem. Biophys. Res. Commun..

[45] Tong Y., Li Y., Gu H., Wang C., Liu F., Shao Y., Li F. (2018). HSF1, in association with MORC2, downregulates ArgBP2 via the PRC2 family in gastric cancer cells. Biochim. Biophys. Acta Mol. Basis Dis..

[46] Liu J., Shao Y., He Y., Ning K., Cui X., Liu F., Wang Z., Li F. (2019). MORC2 promotes development of an aggressive colorectal cancer phenotype through inhibition of NDRG1. Cancer Sci..

[47] Wang T., Qin Z.Y., Wen L.Z., Guo Y., Liu Q., Lei Z.J., Pan W., Liu K.J., Wang X.W., Lai S.J. (2018). Epigenetic restriction of Hippo signaling by MORC2 underlies stemness of hepatocellular carcinoma cells. Cell Death Differ..

[48] Peinado H., Olmeda D., Cano A. (2007). Snail, Zeb and bHLH factors in tumour progression: An alliance against the epithelial phenotype?. Nat. Rev. Cancer.

[49] Kang Y., Siegel P.M., Shu W., Drobnjak M., Kakonen S.M., Cordon-Cardo C., Guise T.A., Massague J. (2003). A multigenic program mediating breast cancer metastasis to bone. Cancer Cell.

[50] Tran H.D., Luitel K., Kim M., Zhang K., Longmore G.D., Tran D.D. (2014). Transient SNAIL1 expression is necessary for metastatic competence in breast cancer. Cancer Res..

[51] Batlle E., Sancho E., Franci C., Dominguez D., Monfar M., Baulida J., Garcia De Herreros A. (2000). The transcription factor snail is a repressor of E-cadherin gene expression in epithelial tumour cells. Nat. Cell Biol..

[52] Chien W., O’Kelly J., Lu D., Leiter A., Sohn J., Yin D., Karlan B., Vadgama J., Lyons K.M., Koeffler H.P. (2011). Expression of connective tissue growth factor (CTGF/CCN2) in breast cancer cells is associated with increased migration and angiogenesis. Int. J. Oncol..

[53] Chen P.S., Wang M.Y., Wu S.N., Su J.L., Hong C.C., Chuang S.E., Chen M.W., Hua K.T., Wu Y.L., Cha S.T. (2007). CTGF enhances the motility of breast cancer cells via an integrin-alphavbeta3-ERK1/2-dependent S100A4-upregulated pathway. J. Cell Sci..

[54] Liu H.Y., Liu Y.Y., Zhang Y.L., Ning Y., Zhang F.L., Li D.Q. (2022). Poly(ADP-ribosyl)ation of acetyltransferase NAT10 by PARP1 is required for its nucleoplasmic translocation and function in response to DNA damage. Cell Commun. Signal..

[55] Yang F., Xie H.Y., Yang L.F., Zhang L., Zhang F.L., Liu H.Y., Li D.Q., Shao Z.M. (2020). Stabilization of MORC2 by estrogen and antiestrogens through GPER1- PRKACA-CMA pathway contributes to estrogen-induced proliferation and endocrine resistance of breast cancer cells. Autophagy.

[56] Hu S.Y., Qian J.X., Yang S.Y., Andriani L., Liao L., Deng L., Huang M.Y., Zhang Y.L., Zhang F.L., Shao Z.M. (2023). Destabilization of microrchidia family CW-type zinc finger 2 via the cyclin-dependent kinase 1-chaperone-mediated autophagy pathway promotes mitotic arrest and enhances cancer cellular sensitivity to microtubule-targeting agents. Clin. Transl. Med..

[57] Zhang S., Guo A., Wang H., Liu J., Dong C., Ren J., Wang G. (2024). Oncogenic MORC2 in cancer development and beyond. Genes. Dis..

[58] Ding Q.S., Zhang L., Wang B.C., Zeng Z., Zou X.Q., Cao P.B., Zhou G.M., Tang M., Wu L., Wu L.L. (2018). Aberrant high expression level of MORC2 is a common character in multiple cancers. Hum. Pathol..

[59] Liao X., Liu C., Ding Z., Wang C., He J., Wu S. (2023). High expression of MORC2 predicts worse neoadjuvant chemotherapy efficacy in triple negative breast cancer. Medicine.

[60] Zhang J., Yang Y., Dong Y., Liu C. (2022). Microrchidia family CW-type zinc finger 2 promotes the proliferation, invasion, migration and epithelial-mesenchymal transition of glioma by regulating PTEN/PI3K/AKT signaling via binding to N-myc downstream regulated gene 1 promoter. Int. J. Mol. Med..

[61] Yang F., Sun R., Hou Z., Zhang F.L., Xiao Y., Yang Y.S., Yang S.Y., Xie Y.F., Liu Y.Y., Luo C. (2022). HSP90 N-terminal inhibitors target oncoprotein MORC2 for autophagic degradation and suppress MORC2-driven breast cancer progression. Clin. Transl. Med..

[62] Saroha H.S., Kumar Guddeti R., Jacob J.P., Kumar Pulukuri K., Karyala P., Pakala S.B. (2022). MORC2/beta-catenin signaling axis promotes proliferation and migration of breast cancer cells. Med. Oncol..

[63] Liao G., Liu X., Wu D., Duan F., Xie X., Wen S., Li Y., Li S. (2019). MORC2 promotes cell growth and metastasis in human cholangiocarcinoma and is negatively regulated by miR-186-5p. Aging.

[64] Pan Z., Ding Q., Guo Q., Guo Y., Wu L., Wu L., Tang M., Yu H., Zhou F. (2018). MORC2, a novel oncogene, is upregulated in liver cancer and contributes to proliferation, metastasis and chemoresistance. Int. J. Oncol..

[65] Wang G., Song Y., Liu T., Wang C., Zhang Q., Liu F., Cai X., Miao Z., Xu H., Xu H. (2015). PAK1-mediated MORC2 phosphorylation promotes gastric tumorigenesis. Oncotarget.

[66] Zhang F.L., Cao J.L., Xie H.Y., Sun R., Yang L.F., Shao Z.M., Li D.Q. (2018). Cancer-Associated MORC2-Mutant M276I Regulates an hnRNPM-Mediated CD44 Splicing Switch to Promote Invasion and Metastasis in Triple-Negative Breast Cancer. Cancer Res..

[67] Liberti M.V., Locasale J.W. (2016). The Warburg Effect: How Does it Benefit Cancer Cells?. Trends Biochem. Sci..

[68] Hanahan D., Weinberg R.A. (2011). Hallmarks of cancer: The next generation. Cell.

[69] Vander Heiden M.G., Cantley L.C., Thompson C.B. (2009). Understanding the Warburg effect: The metabolic requirements of cell proliferation. Science.

[70] Guddeti R.K., Pacharla H., Yellapu N.K., Karyala P., Pakala S.B. (2023). MORC2 and MAX contributes to the expression of glycolytic enzymes, breast cancer cell proliferation and migration. Med. Oncol..

[71] Sanchez-Solana B., Li D.Q., Kumar R. (2014). Cytosolic functions of MORC2 in lipogenesis and adipogenesis. Biochim. Biophys. Acta.

[72] Su Y., Yu T., Wang Y., Huang X., Wei X. (2021). Circular RNA circDNM3OS Functions as a miR-145-5p Sponge to Accelerate Cholangiocarcinoma Growth and Glutamine Metabolism by Upregulating MORC2. Onco Targets Ther..

